# Implementation of a Technology-Based Mobile Obstetric Referral Emergency System (MORES): Qualitative Assessment of Health Workers in Rural Liberia

**DOI:** 10.2196/58624

**Published:** 2024-11-13

**Authors:** Christopher W Reynolds, HaEun Lee, Joseph Sieka, Joseph Perosky, Jody R Lori

**Affiliations:** 1 University of Michigan Medicine Ann Arbor, MI United States; 2 University of Michigan School of Nursing Ann Arbor, MI United States; 3 A.M. Dogiloti College of Medicine University of Liberia Monrovia Liberia; 4 Spectrum Health Grand Rapids, MI United States

**Keywords:** mHealth, mobile triage, referral pathways, Liberia, LMIC, low- income country, obstetric triage, third delay, mobile health, mobile application, digital health, digital intervention, smartphone, middle-income country

## Abstract

**Background:**

Maternal mortality remains a persistent challenge in low- and middle-income countries, where evidence-based interventions of obstetric triage and prehospital communication remain sparse. There is limited implementation evidence for technology-based approaches to improve obstetric care in such contexts. Liberia struggles with maternal mortality, particularly in rural areas where deaths are attributable to delays from absent triage and interfacility communication. We implemented a Mobile Obstetric Referral Emergency System (MORES) in rural Bong County to improve prehospital transfer, health worker attentiveness, and patient care for critical obstetric patients. MORES consisted of triage training and a 2-way, templated WhatsApp communication system to reduce delays among patients transferred from rural health facilities (RHF) to hospitals.

**Objective:**

This study aimed to examine MORES implementation outcomes of usability, fidelity, effectiveness, sustainability, and scalability, as well as additional impacts on the wider health system.

**Methods:**

A structured case study design interview was developed by Liberian and US experts in obstetric triage. Participants included 62 frontline obstetric health providers including midwives (38/62, 61%), nurses (20/62, 32%), physicians assistants (3/62, 5%), and physicians (1/62, 2%) from 19 RHFs and 2 district hospitals who had used MORES for 1 year. Individual interviews were conducted on MORES implementation outcomes, transcribed, and analyzed in NVivo (version 12; Lumivero) with a team-based coding methodology. Content analysis with a deductive approach examined implementation outcomes of usability, fidelity, effectiveness, sustainability, and scalability, while an inductive approach categorized the unanticipated impacts of MORES on the wider health system.

**Results:**

Four domains were identified regarding MORES implementation: Usability and Fidelity, Effectiveness, Sustainability and Scalability, and Health System Impact. All participants perceived MORES to have high usability and fidelity, as the triage and messaging system was implemented as intended for critical obstetric patients (62/62, 100%). For effectiveness, MORES accomplished its intended aims by improving prehospital transfer (57/62, 92%), increasing health worker attentiveness (39/62, 63%), and contributing to improved patient care (34/62, 55%). MORES was perceived as sustainable and scalable (62/62, 100%), particularly if technological barriers (21/62, 34%) and staff training (19/62, 31%) were addressed. MORES impacted the wider health system in unanticipated ways including improved coordination and accountability (55/62, 89%), feedback mechanisms for hospitals and RHFs (48/62, 77%), interprofessional teamwork (21/62, 34%), longitudinal follow-up care (20/62, 32%), creating a record of care delays (17/62, 27%), and electronic health record infrastructure (13/62, 21%).

**Conclusions:**

MORES was perceived to have high usability, fidelity, effectiveness, sustainability, and scalability by frontline obstetric providers in rural Liberia. MORES accomplished the intended aims of improving prehospital transfer, increasing health worker attentiveness, and contributing to improved patient care. Additionally, MORES strengthened the health system through 6 domains which impacted individual and system levels. Future studies should quantitatively evaluate delay and morbidity reductions and strategies for scaling MORES.

## Introduction

Despite increased attention, funding, and innovation, maternal mortality continues to disproportionately affect low- and middle-income countries (LMICs) [1]. Liberia in West Africa has one of the highest maternal mortality ratios in the world with 652 deaths per 100,000 births [2]. This inequity is due to many factors including resource limitations, workforce shortages, and an overall unstable and underfunded health system following decades of conflict, the 2014-2016 Ebola epidemic, and COVID-19 pandemic [3]. A major contributing factor impacting Liberia’s high maternal mortality is a lack of standardized obstetric triage protocols and prehospital communication systems between health facilities [4]. Such systems allow for efficient patient transfer and have consistently been shown to improve maternal and neonatal health outcomes [5,6]. In Liberia, a lack of standardized triage, referral, and transfer systems leads to care delays and increases morbidity at the prehospital and intra-facility level, also known as the “third delay,” which occurs after patients arrive at health facilities [7]. This lack of infrastructure creates a critical blind spot for timely maternal care, especially for patients in rural areas who must travel hours on foot or by personal car to reach a health facility.

Over the last decade, the Liberian Ministry of Health has emphasized the importance of combating maternal and perinatal mortality at community and national levels [8]. Rural health facilities (RHFs) are community clinics established throughout rural areas of Liberia to serve as the first line of care for patients living far from district hospitals [9]. RHF staff, midwives, and nurses are the frontline obstetric health workers in Liberia, responsible for preliminary assessments, treatment and stabilization, and escalation of care by referring patients to district hospitals when necessary [10]. However, care delays persist due to system limitations. Verbal autopsy reports of 35 maternal deaths from Bong County, Liberia revealed 2 major contributors to maternal death: inadequate staff training and ineffective communication between RHFs and hospitals [4]. A promising solution to improve maternal transfer and prehospital communication systems leverages mobile health interventions (mHealth), including messaging platforms to transmit patient information from a referring facility to the hospital prior to arrival [11]. Such systems may be a promising intervention to reduce prehospital and intrafacility delays by allowing health workers to prepare resources for immediate intervention upon patient arrival [12]. Several mHealth approaches have been successful in reducing care delays in LMICs including Ghana, and a recent study by our team showed high desirability for such a program among 130 health workers from RHFs and hospitals in Bong County [13,14].

In response, our team developed and implemented the Mobile Obstetric Referral Emergency System (MORES) among 20 RHFs and 2 district referral hospitals in Bong County, Liberia. MORES has two main components as follows: (1) triage capacity building training among obstetric health workers, and (2) implementation of a mobile, WhatsApp-based communication platform, aimed at reducing prehospital and intrafacility care delays [15]. Specifically, the intended aims of MORES were to reduce care delays through improved prehospital transfer, increased health worker attentiveness, and improved patient care. MORES works in the following ways: when a pregnant patient presents to an RHF in critical condition, she will undergo standardized triage by a community nurse midwife to assess acuity. If the RHF midwife determines that this patient requires higher level or urgent care at the district hospital, the RHF midwife will send a templated WhatsApp message to the district hospital with information including the patient’s condition, past medical and obstetric history, any interventions administered, and method of transport to the hospital. Immediately upon receiving this message from the RHF midwife, hospital obstetric nurses begin preparing for that patient’s arrival including gathering needed medications, informing physicians, readying the emergency department, and preparing the operating room if urgent surgical intervention is anticipated. If delays emerge during patient transport, the RHF and facility providers can work collaboratively through MORES messaging to overcome these barriers. Upon arrival at the hospital, the referred obstetric patient will undergo an additional standardized triage by the obstetric nurses and receive a green, yellow, or red wristband corresponding to acuity level before admission or initiation of urgent intervention. Implemented in March 2022, MORES demonstrated significant improvements in triage knowledge and was used by more than 50 health workers to send 359 referral messages to transfer patients over the course of a year.

In this qualitative study, we evaluated the implementation outcomes of MORES including usability, fidelity, effectiveness of accomplishing intended aims, sustainability, and scalability. We also evaluated if MORES had additional impacts on the health system more broadly beyond its intended impacts.

## Methods

### Study Setting

This study took place in Bong County, Liberia, the third most populous and primarily rural county in Liberia [14]. Researchers at the University of Liberia and the University of Michigan partnered with the Bong County Health Team to design and implement MORES. Further detail of the MORES intervention, including its impact on health outcomes, is published elsewhere [15]. This study was conducted 1 year post implementation. Bong County was selected as the study site following efforts by the Liberia Ministry of Health to focus on improving maternal outcomes in this region given recent increases in maternal deaths [4]. Additionally, we have strong institutional partnerships between researchers and the County Health Team. Prior to MORES, Bong County had limited prehospital infrastructure consisting of 2 ambulances, no medical dispatch, and limited avenues for RHFs to communicate with hospitals. Health workers previously documented all medical information on a referral paper, which the patient was responsible for carrying and presenting to the referral hospital upon arrival. Often taking hours to arrive at said hospital, these papers would frequently be lost or damaged in transit.

### Mores Implementation

In March 2022, specialists from Ghana and the University of Michigan with extensive experience in mHealth prehospital obstetric systems conducted capacity-building training among health workers from 20 RHFs and 2 district hospitals. Details regarding the templated communication messages and initial MORES training have been described [15]. Following this training, health workers returned to their respective health facilities to train colleagues in MORES. The 2 district hospitals were provided with smartphones to be used in the emergency department solely for MORES, while RHF workers used their personal cell phones. RHF and hospital workers were supplied cellular scratch cards to reload data at regular intervals throughout the study.

### Study Design

To determine health worker perceptions of MORES implementation outcomes, we conducted a qualitative study using content analysis, following 1 year of MORES implementation in Bong County. First, US and Liberian experts in obstetric care and qualitative methods developed a structured interview script. The interview script was reviewed with Liberian health workers to adjust the language to the local context, purge repetitive items, and consolidate questions. The final interview script contained 20 items evaluating 6 major metrics including demographics, MORES purpose, usability, impact on referrals, facility linkages and feedback, and areas of strength and improvement (Multimedia Appendix 1).

### Data Collection and Participants

In March 2023, 10 research assistants (RAs) from the University of Liberia traveled to Bong County to conduct interviews. The RAs consisted of both male and female Master of Public Health Students who had completed their course of study and were working on their Master theses and Master students who had completed a qualitative methods course. All were bachelor degree holders in various health-related fields and had extensive prior experience conducting qualitative interviews, which was a criterion for RA selection. During the data collection period, participants knew that RAs were students from the University of Liberia with an interest in obstetric health access and part of the research team who would be studying the implementation of MORES within their health system. A prior relationship with the study team had been established, given that this group was the same that implemented MORES 1 year prior.

In collaboration with the Bong County Health Team, RAs invited all health workers from the 20 RHFs and 2 district hospitals who had used MORES in the past year to participate using purposive sampling. Health workers were eligible if they were 18 years or older, currently employed by a Bong County health facility, and had used MORES at least once. Participants were excluded if they were unable to allocate sufficient time for an uninterrupted interview (10 minutes) or had never used MORES. RAs approached eligible participants face-to-face and invited them to complete the interview. Written consent was collected from eligible health workers who were willing to participate. It was made clear that the interview was anonymous, voluntary, and that their answers or decision of whether to participate would not influence their employment or ability to use MORES in the future. Individual interviews were conducted in a private location at the RHF or hospital according to participant preference. Each interview took approximately 10 minutes, and the interviews were audio recorded and stored in a secure lock box for transport to the University of Liberia. Researchers from Liberia and the University of Michigan met periodically throughout the data collection period to determine data saturation. Though saturation occurred after approximately 30 interviews, the opportunity to participate remained available so that all health workers who wished to share their perceptions were able to.

### Data Analysis

Interviews were transcribed in Microsoft Word and immediately uploaded to a secure Dropbox folder only available to study researchers. All identifiable information was omitted during transcription. Two researchers began analysis through iterative immersion in the data, using content analysis with a deductive approach to categorize implementation outcomes of usability, fidelity, effectiveness, sustainability, and scalability to guide codebook development. Upon immersion in the data, it was determined that additional, unanticipated themes of MORES impact on the wider health system were frequently mentioned throughout the interviews. Therefore, a combined deductive-inductive content analysis approach was used to generate codes that categorized study aims while also accounting for the emergence of new themes. Once domains and major themes were identified, the codebook was further refined to identify subthemes under the major themes [16]. Transcripts were imported to NVivo (version 12; Lumivero) and coded using the codebook with a content analysis approach to answer the research question: “What are health workers’ perceptions on the implementation of MORES in light of its intended aims and impact on the wider health system?” [17]. Multiple measures were taken to ensure trustworthiness including (1) triangulation of interviews from multiple health specialists, (2) creation and validation of a consensus codebook, (3) inclusion of Liberian health workers as core members of the analytic team, and (4) member checking with Liberian health workers regarding key results.

### Ethical Considerations

This study was approved by the University of Michigan institutional review board (HUM00195449) and the University of Liberia institutional review board (IRB00013730), whose protocols were followed throughout the entire study period. All participants underwent a process of written, informed consent prior to participation and all data were deidentified prior to analysis. This study adheres to COREQ (consolidated criteria for reporting qualitative research) guidelines for reporting in qualitative research [18].

## Results

### Participant Demographics

**Figure 1:**
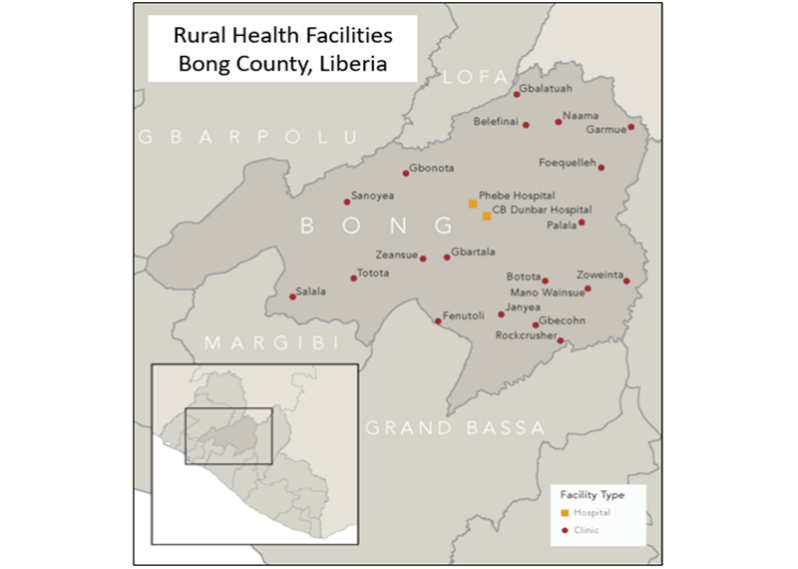
Rural health facilities and hospitals participating in the Mobile Obstetric Referral Emergency Systems (MORES) in Bong County, Liberia. Hospitals are denoted in yellow (2), with RHFs in red (19).

Sixty-two health workers from 19 RHFs and 2 district hospitals participated, representing 95% of total facilities taking part in MORES (Figure 1). Health workers from one facility included in the original training did not transmit any messages due to the lack of a smartphone with WhatsApp capability, therefore health workers from 19 facilities were included in our data collection. Participants included registered nurses, midwives, physician assistants, and medical doctors (Table 1). Three participants had additional specialization as neonatal specialists, and 12 occupied leadership roles, including nursing officer-in-charge (n=9), obstetrics supervisor (n=2), and chief medical officer (n=1). Most participants were between 35 and 45 years old, with years of professional experience ranging from 1 to 19 years (median 8 years, IQR 5-10). Across facilities, participants working in hospitals were slightly older and had more work experience than those in RHFs.

**Figure 2:**
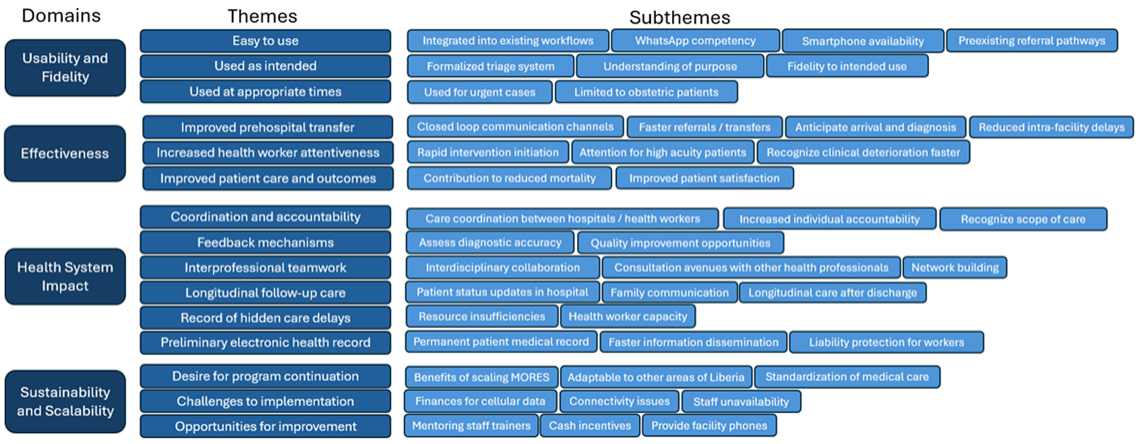
Organization of domains, themes, and subthemes from MORES interviews. MORES: Mobile Obstetric Referral Emergency System.

Four major domains were used to evaluate the implementation of MORES: (1) usability and fidelity, (2) effectiveness of intended impact, (3) sustainability and scalability, and (4) health system impact. Among these domains were 15 themes and 43 subthemes, outlined in Figure 2.

**Table 1:** Participant demographics for the Mobile Obstetric Referral Emergency System (MORES) evaluation study in Bong County, Liberia.

			Total (N=62)		Rural health facilities (n=33)		Hospital 1 (CB Dunbar) (n=15)		Hospital 2 (Phebe) (n=14)
**Age (years), n (%)**									
	<20	0 (0)		0 (0)		0 (0)		0 (0)	
	20-29	6 (10)		5 (15)		0 (0)		1 (7)	
	30-39	30 (48)		19 (58)		7 (47)		4 (29)	
	40-49	17 (27)		9 (27)		5 (33)		3 (21)	
	>50	7 (11)		0 (0)		2 (13)		5 (36)	
	Unknown	2 (3)		0 (0)		1 (7)		1 (7)	
Age (years), mean (SD)			39.2 (7.22)		36.7 (4.96)		42.1 (6.91)		42.7 (9.46)
**Role, n (%)**									
	Registered nurse	20 (32)		10 (30)		5 (33)		5 (36)	
	Midwife	38 (61)		23 (70)		9 (60)		6 (43)	
	Physician assistant	3 (5)		0 (0)		1 (7)		2 (14)	
	Medical doctor	1 (2)		0 (0)		0 (0)		1 (7)	
**Time in practice, n (%)**									
	<3 years	4 (6)		4 (12)		0 (0)		0 (0)	
	3-6 years	19 (31)		13 (39)		2 (13)		4 (29)	
	7-10 years	24 (39)		12 (36)		7 (47)		5 (36)	
	11-15 years	11 (18)		3 (9)		4 (27)		4 (29)	
	16-20 years	2 (3)		1 (3)		1 (7)		0 (0)	
	Unknown	2 (3)		0 (0)		1 (7)		1 (7)	
Time in practice (years), mean (SD)			7.8 (3.61)		6.7 (3.60)		9.6 (3.20)		8.5 (3.10)

### MORES Usability and Fidelity

MORES was evaluated for its usability and fidelity, meaning if participants used MORES as it was intended to be implemented. For usability, all 62 participants affirmed the MORES intervention was easy to use and beneficial to the patients they served (100%). They described MORES as easily integrated into existing workflows within their health system, as most had access to smartphones, knew how to use WhatsApp, and used MORES as part of a preexisting RHF-hospital referral system. For fidelity, participants described how they used MORES as it was intended to be implemented, including for making patient referrals, interfacility communication, and triage upon hospital arrival: “We get the patient’s name, age, reasoning of referral, mode of transfer. If referring through an ambulance or commercial car, we can place it in the WhatsApp group chat and send it” [P41].

Upon arrival at the hospital, patients were immediately triaged with a green, yellow, or red wristband according to acuity: “The band is there to help us to separate patients according to the conditions. The red is a critical condition; yellow is for someone that has a bad condition but not very critical; and the green is for normal patients” [P29]. Bands were worn until discharge, with health workers continually monitoring patients for changes in the category and exchanging wristbands appropriately.

All participants expressed that they used MORES for acutely sick patients at appropriate times. For hospitals, this was multiple times per day, given their high volume: “We use it all of the time, when the patient arrives in the facility. Any patient that comes in the labor room or the entry point, the ER” [P15]. At RHFs with smaller catchment areas, rates of emergency presentation were less frequent: “Sometimes in a month, we can get 4-6 [emergencies]. All depends on how the patients will come” [P31].

There was a clear understanding of the purpose of MORES from all providers and facilities, as every participant (62/62, 100%) accurately defined at least 1 goal of the program:

[MORES] is a quick emergency guide that will inform you where to refer, because you put the patient’s information on the WhatsApp platform and send it to the referral site. They will receive it and be preparing for the patient

P56

### MORES Effectiveness

MORES was evaluated for effectiveness, meaning whether or not the program accomplished its intended purpose. These goals included improving prehospital transfer, increasing health worker attentiveness, and improving patient care and outcomes. Nearly all participants reported improved prehospital transfer (57/62, 92%). A major factor for improved transfer was that MORES established an avenue for rapid, prehospital communication between RHF and hospital health professionals, which facilitated closed-loop communication:

If [RHFs] communicate that they are sending a patient and we reply, ‘yes, we’re awaiting the patient’ then they are aware that we have received the message. Then we have to give them feedback that the patient came and arrived at this time. We send information like the patient came with this kind of condition and we’ve started management or the patient is improving or the patient is on treatment. Until that patient can be discharged, we still have to give them feedback.

P1

Participants believed that this communication resulted in more timely referrals from RHFs to hospitals (53/62, 85%). MORES also allowed health professionals to collaboratively overcome referral and transport facilitation barriers and informed clinicians on patient arrival times and conditions: “Before, people were not there to receive the patient on time. But now, if you send information, they will already be standing at the entrance waiting to receive the patient” [P48]. This led to a reduction in transfer time and intrafacility, or third delays, at hospitals, which was especially important for urgent cases requiring surgical intervention: “It has helped us limit delays from patients being referred. It informs the ER and OB Ward in times of emergency and gives us prior notice about a referred patient that has limited time for surgery” [P21].

Participants reported being able to prepare operating rooms and gather needed materials for urgent intervention before patient arrival, to more quickly evaluate and treat critically ill patients upon arrival: “As a health worker, it’s helpful in the sense that before my patient arrives, I’ve already received their information and setup. The moment they arrive, immediate intervention starts” [P6].

Second, health workers believed that MORES increased their attentiveness toward patients (39/62, 63%). One stated “It’s useful because it helps to keep us focused and makes us know that there’s an emergency that you need to act right away” [P9]*.* Another agreed that MORES trained her to recognize clinical deterioration faster: “Most often, if a patient comes, I will already know and be on guard to monitor because any time the patient’s condition can change” [P7]*.*

This attentiveness was partially attributed to improved triage capacity following the triage training sessions and wristband implementation (35/62, 56%). One hospital health worker described the case of a woman with peripartum hemorrhage who received timely care because of the triage system. Another affirmed that the triage wristbands helped focus health workers’ urgency, with patients with red wristbands being seen first on clinician rounds (Table 2).

Finally, more than half of the participants (34/62, 55%) believed that MORES contributed to improving patient outcomes. They perceived MORES as helping to improve maternal and neonatal mortality, as well as patient satisfaction:

It has helped me to save lives that were to be lost, especially in pregnant women cases. If someone has a ruptured ectopic, before the person comes, we already know the person’s condition…and the person will receive the care in time.

P14

**Table 2:** Effectiveness domain: themes, subthemes, and representative quotes.

Theme and subtheme		Representative quote
**Theme 1: Improved prehospital transfer (57/62, 92%)**		
	Closed loop communication channels	*It provides information ahead of time to inform a bigger facility that a patient is coming with their diagnosis. It allows for information dissemination.* [P50]
	Faster referral and transfer times	*It helps reduce the time if you’re referring a patient. You have to give the patient’s information, then the health worker, doctor, or midwife will be able to know the condition that is coming and prepare before time to avoid delays.* [P34]
	Anticipate patient arrival and diagnosis	*If the RHFs have any critical patients, they will send a message before the patient reaches the hospital, and we will already know the type of patient that is coming and their condition so that we can get prepared for the patient.* [P18]
	Reduced intrafacility delays through materials preparation and intervention initiation	*When we know the case coming, we will setup and get prepared. Whatsoever materials that they will need like drugs, the team will get everything set as they arrive, so we just start our care. No more going to look for MgSO4 for an eclamptic patient. You get your tray set so as soon as they enter, you start treatment.* [P1]
**Theme 2: Increased health worker attentiveness (39/62, 63%)**		
	Formalized and improved triage system	*[MORES] improves care quality because when the patients come to the emergency room and they are already triaged, they will place the band on the patients’ hands to know the kind of patients so everyone will be on the guard to monitor the patients.* [P7]
	Increased attention for high acuity patients	*The moment the doctors enter the emergency room (ER), they go directly to the patients with the red band because they are patients of concern. It provides information on which patient to see first.* [P6]
	Recognize patient deterioration or complications faster	*Staff that is on shift will receive the patient and label them for the next person who is coming. As soon the person enters the place and sees the patients, they will know who to care for first; who to start with or who’s the patient of concern. Patients with red are our patients of concern. If green, you know it’s a normal patient; someone who’s in yellow will require attention because they can change to red.* [P1]
**Theme 3: Improved patient care and outcomes (34/62, 55%)**		
	Contribution to reducing mortality	*It helps the pregnant woman to be well taken care of and to help save their lives and that of the babies.* [P29]
	Patient satisfaction	*Because information is disseminated and prompt referral is given before patient arrival, it makes patients happy.* [P50]

### MORES Sustainability and Scalability

Interview responses reflected an overwhelmingly positive desire for MORES to continue and expand (62/62, 100%): “My recommendation is for the program to continue because it’s really helpful to us and reduces our maternal deaths” [P35]*.* Participants discussed the benefit of scaling MORES to other RHFs, who: “complained on why they are not part of the program,” and citing that, “there are some terrible cases that come from the other facilities that are not trained” [P6]. They believed benefits could be realized in standardization of medical care, reduction in error, and ultimately improvement in maternal and neonatal mortality if the program was scaled to other counties in Liberia. Participants directly cited MORES usability, feasibility, and effectiveness as justification for its scaling potential.

Despite successes, there were ongoing challenges to be addressed prior to scale-up, with 89% (n=55) of participants naming at least 1 issue. Challenges included financial support for cellular data (39/62, 63%), poor cellular networks preventing connectivity (21/62, 34%), and staff unavailability due to high turnover and limited motivation (19/62, 31%). RHF health workers were more likely to mention poor cellular networks than hospital staff, who worked in urban and suburban settings. Three participants also explained how conflict could arise between patient care and MORES, particularly when checking messages on a busy service: “Especially at the emergency unit, sometimes we are busy and forget to check the WhatsApp phone. If you have an emergency, you will be busy with that while other clinics are sending messages.” [P10].

From these challenges, participants offered suggestions for improvement. For workforce shortages, participants encouraged mentoring more staff to be MORES trainers to onboard new health workers: “If the knowledge of nurses can be increased and incorporating more nurses and increasing the training method, it will help support the program.” [P21]*.* Others suggested increasing staff motivation through cash incentives and providing facility phones to all RHFs with mechanisms for data recharging, rather than relying on personal phone use.

### MORES Health System Impact

**Figure 3:**
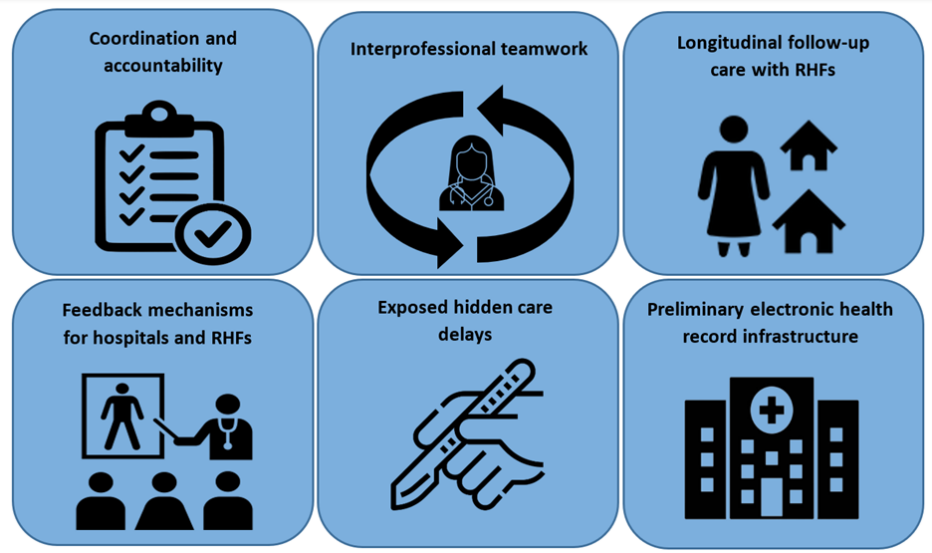
Six themes of health systems strengthening which emerged from the implementation of MORES according to participant interviews. MORES: Mobile Obstetric Referral Emergency System; RHF: rural health facility.

In addition to evaluating MORES implementation based on intended aims, there were multiple effects of MORES on the wider health system that were not part of the original design. In total, 61 (98%) participants described ways that MORES implementation strengthened aspects of the health system beyond the intended aims of implementation. Participants identified 6 ways in which MORES contributed to broader health systems strengthening (Figure 3). First, participants noted increased coordination and accountability among health workers and facilities when caring for patients (55/62, 89%). This was perceived through rapidly updated information and the ability to coordinate care between receiving hospitals which previously had limited communication:

I appreciate the program because care is on time these days and people are aware. When a patient is coming but C.B Dunbar is crowded and there’s no space, as long we get the message, we send it to Phebe for preparations. It helps a lot.

P1

Coordination and accountability were also experienced at a personal level, with health workers reporting increased responsibility to their patients, including their scope of care: “[MORES] got us to be on our guard and to know which case is above our limit and needs to be transferred to a bigger facility” [P54]. Workers felt more comfortable acknowledging personal limitations and used the MORES system to consult higher-level colleagues more frequently than prior.

Second, feedback mechanisms for hospitals and RHFs were a key benefit of the 2-way WhatsApp messaging (48/62, 77%). Hospital staff offered constructive feedback to RHF workers on their care and referral process. This feedback included the accuracy of diagnoses and opportunities for quality improvement:

Based on the feedback, we will know that we are doing good work or understanding the referral system. Using the paper-based system, getting feedback used to be difficult, but now it comes as soon as the patient arrives

P37

Feedback was rapid, with 89% of RHF health workers receiving updates within the first 24 hours (n=55). Such communication also facilitated improved interprofessional teamwork (21/62, 34%). Participants reported reduced work tensions between RHF and facility providers and improved interdisciplinary collaboration through network building: “It is helpful because my community health assistants and midwives are working together. If a patient is not willing to come, they will continue encouraging the patient” [P40]*.* RHF health workers appreciated that they could consult dozens of other professionals when managing complex patients, establishing consultation avenues for health workers who are often the sole clinicians working in a low-resource facility, and learning from the outcomes of one another: “I use it to refer, but also see if my colleagues are using it, because we all are trying to stop the maternal death rate in Liberia. I use it to see the outcome of my colleagues’ referral” [P47].

Fourth, MORES facilitated longitudinal follow-up care for RHFs (20/62, 32%), especially through patient tracking while still in the hospital. These updates helped family members unable to visit loved ones: “It makes us know more about our patients because the hospital will give [updates]. If the patient’s relatives don’t have a phone, we will be able to inform them on the status” [P35]. Hospitals additionally used MORES to communicate discharge instructions to RHFs for patients requiring follow-up. One RHF midwife elaborated on how MORES empowered her to help a patient continue her hospital-prescribed medication following discharge (Table 3).

**Table 3:** Health system impact domain: themes, subthemes, and representative quotes.

Theme and subtheme		Representative quotes
**Theme 1: Coordination and accountability (55/62, 89%)**		
	Care coordination between hospitals and health workers	*When the patient comes as an emergency, if we call and can’t get the ambulance, as soon as we send the information the people at Phebe will inform the ambulance team to come for the patient.* [P35]
	Increased individual accountability	*Before, there was conflict. Midwives would delay the patients and write referral notes to suit their taste while patients were saying something different. No one was willing to take the blame.* [P24]
	Recognize scope of care	*It’s very helpful because we don’t have to keep cases that we know we can’t manage. Once they come to the facility, if the case is in our reach, we can handle it, but if not, we refer them.* [P41]
**Theme 2: Feedback mechanisms for hospitals and RHFs (48/62, 77%)**		
	Identify quality improvement opportunities	*It’s helpful. Sometimes we get negative feedback from the patients, like if a patient gives birth and loses her baby.* [P49]
	Assess diagnostic accuracy	*You are doing work and get feedback. It can help you in your diagnosis because if the people at the hospital diagnose the same thing, you can be confident of yourself* [P30]
**Theme 3: Interprofessional teamwork (21/62, 34%)**		
	Interdisciplinary collaboration	*Using [MORES], I will be in communication with hospital nurses at CB Dunbar or Phebe to know if the patient is in good condition or if there is something that should make me concerned.* [P38]
	Consultation avenues with other health workers	*The program is good; it gives information. Whatever happens in Palala Clinic is being communicated in all facilities and we are aware of all our patients.* [P50]
	Network building	*It helps us do plenty things. It makes us to know lot of people and how to really deal with the phone. It also makes us to know every patient information that needs to be communicated.* [P42]
**Theme 4: Longitudinal follow-up care with RHFs (20/62, 32%)**		
	Patient status updates in the hospital	*It makes us help our patients get quick health services at the higher facilities and for us to get information on how they are doing.* [P35]
	Communication with family members	*The feedback is very helpful because it confirms and updates on the conditions of our patients that we sent. We can use the feedback to tell the patient’s people.* [P46]
	Longitudinal care after discharge	*The feedback helps us continue the treatment or medication of a patient that has been referred [once discharged].* [P58]
**Theme 5: Exposed hidden care delays (17/62, 27%)**		
	Resource insufficiencies	*[We realized we] need emergency materials to make work easier. All OB cases are actually emergency cases and should be treated.* [P22]
	Health worker capacity	*One difficulty we experience is having misdiagnosis from the clinics. Communication sometimes isn’t clear.* [P17]
**Theme 6: Preliminary electronic health record infrastructure (13/62, 21%)**		
	Permanent patient medical record	*[MORES is] helping a lot especially with data [capture]. Patient referral and treatment between facilities is being communicated.* [P54]
	Faster information dissemination	*We now receive electronic communication. It makes us more confident in our work and we feel satisfied after getting our findings.* [P50]
	Liability protection for workers	*It’s very helpful to me because it will save me. If I use WhatsApp to refer my patient, the time I refer will be mentioned. If there’s any delay, they will know where it comes from. So, it will protect me.* [P55]

Fifth, MORES created a record of hidden care delays in Bong County by documenting reasons for delays as they arose (17/62, 27%). The most common included resource insufficiencies and worker capacity: “The purpose of [MORES] is to do quick referral and…to know where the delay comes from; whether from the community, the relatives or the midwife or from the county because they are responsible to send the ambulance” [P55]. In such situations, even when MORES worked properly, patients still experienced delays. More than half of respondents listed factors outside of MORES that worsened care delays for patients (35/62, 56%), including a lack of medications, intravenous fluids, and ambulance insufficiency.

Finally, MORES benefited the health system by creating a preliminary electronic health record (EHR) infrastructure (13/62, 21%). No EHR exists in Bong, and all documentation occurs on paper charts. Participants appreciated the ease, accessibility, and speed that MORES provided, believing it sped information dissemination:

[MORES] enables me to do my work with ease. The time I take to write a whole lot of papers…wastes the patient’s time, and it will also waste time at the referral hospital…With [MORES], the hospital will already get the information before the patient can arrive so they will start to attend immediately.

P47

This EHR also started a permanent record of patient conditions, transfers, and treatments administered, so that health teams could review patients requiring special accommodations or difficult cases. Finally, health workers appreciated the protections that having a permanent record of electronic communication offered in terms of liability for malpractice.

## Discussion

### Principal Findings

From this qualitative study of frontline health workers in rural Liberia, we found that implementation of the MORES intervention was perceived as having high usability, fidelity, and effectiveness for its intended aims. Furthermore, MORES had the unanticipated impact of strengthening multiple components of the health system and demonstrated exciting potential for scaling and sustainability. These findings confirmed the results of our preintervention study which showed high desirability and acceptability of the WhatsApp-based triage, transfer, and referral system among rural Liberian health workers to better care for obstetric patients [14]. Our analysis further revealed health worker perceptions regarding the benefits and health system improvement opportunities through MORES. These in-depth results contribute to the findings from other studies on the impact of MORES, including increased rates of cesarean section and lower odds of nonvigorous symptoms in neonates [15].

Users perceived MORES to be highly usable, as it could be seamlessly integrated into existing workflows and referral pathways. MORES use also demonstrated high fidelity, as it was implemented as intended and at appropriate times to refer critical patients. MORES participants reported the program to be effective across its intended aims: improving prehospital transfer, increasing health worker attentiveness, and improving patient care. The triage training and templated, bidirectional WhatsApp communication components of MORES worked collaboratively to address 2 key gaps in rural care delivery in Liberia: a lack of standardized obstetric triage protocols and a limited prehospital communication system. Increasing capacity and infrastructure to support triage protocols and prehospital emergency systems have been shown to significantly reduce patient morbidity and mortality, particularly in LMICs [19]. This opportunity for impact is especially prevalent for maternal mortality, where the third delay accounts for a massive burden of maternal and neonatal deaths, in some cases as high as 80% [20]. Participants were enthusiastic about the impact of MORES and desired its sustainability and scaling to other health facilities throughout Liberia. Continuing to implement and evaluate programs that increase capacity in obstetric triage, prehospital communication, and facility referral and transfer will be important for improving care and understanding the effects of mHealth technologies in resource-limited environments.

Though not an intended aim of implementing MORES, we found that the program facilitated health systems strengthening across 6 themes: coordination and accountability, feedback mechanisms for hospitals and RHFs, interprofessional teamwork, longitudinal follow-up care with RHFs, a record of hidden care delays, and establishment of a preliminary EHR infrastructure. While not the primary goals of MORES, these effects highlight important benefits across many internal and external domains of health system strengthening. For internal effects, participants reported MORES improved personal accountability, quality improvement opportunities, network building, and reduced workplace tensions and stress from liability. Externally, participants perceived faster information dissemination and facility preparation, consultation mechanisms, and care continuity. These findings highlight how MORES contributed to capacity building and the beneficial effects that can be realized when interventions are implemented to improve systems beyond individual disease states [21]. For example, while MORES was intended for obstetric and peripartum referrals, it exhibited the potential to be adapted to other specialties such as emergency medicine and surgery [22]. MORES provides a pragmatic example of how horizontally implementing interventions across multiple facilities, worker levels, and patient conditions can have multiplicative effects on health systems improvement.

This study adds to the growing literature on mHealth implementation in LMICs to reduce delays and improve care access. One comprehensive review of mHealth interventions for maternal health in LMICs showed that health worker buy-in and motivation, perceived intervention usability, and efficient messaging were key implementation-related mechanisms for success [23]. Our findings on MORES account for all 3, showing high usability and desires for sustainability and scaling, with efficient communication mechanisms that were used appropriately, bidirectionally, and rapidly. A study in South Africa demonstrated high usability for a mobile health platform to aid health workers in the management of pregnant women at risk for preeclampsia [24]. From this program, most user issues resulted from phone features including scroll wheels and touch screens. In MORES, we leveraged the free, WhatsApp communication platform already familiar to Liberian health workers, thereby mitigating user errors from unfamiliarity. Similar to our findings on the impact on interprofessional dynamics, other studies have demonstrated the collaborative effects of a WhatsApp messaging platform among obstetric providers, including as avenues for consultation, network building, and interprofessional communication [25]. Recently in Ghana, a WhatsApp messaging technology was implemented among 13 facilities and 81 health workers for more than 600 patient referrals from rural to district hospitals [26]. Findings from this study mirrored ours, including exposing hidden care delays and creating a preliminary EHR. Specifically, the program identified delay factors, referral characteristics associated with faster transport, and timely updates on patient arrival and outcomes more quickly and permanently compared with paper charting. A mHealth intervention among traditional birth attendants for indigenous women in rural Guatemala demonstrated increased referrals to facility-level care but similar complication rates across intervention and control groups [27]. Such inconclusive outcomes data suggest opportunities for future evaluation of maternal mHealth interventions. Results of a meta-analysis from 2016 revealed that out of 15 articles, only 2 studies showed a low risk of bias and 1 demonstrated a mortality benefit when text messaging women during pregnancy [28]. Future quantitative findings regarding the impact of MORES on maternal outcomes and referral times could clarify the potential clinical impact of well-implemented and highly acceptable maternal mHealth in LMICs.

### Limitations

This study had several limitations. First, our qualitative methods and geographic homogeneity limit the generalizability of results. However, this approach was considered most effective given the study aims which allowed for in-depth explanations of MORES usability, fidelity, and effectiveness. Second, our sample had an unevenly distributed representation of health worker roles, with most participants being midwives and nurses. Increasing recruitment of physicians and other personnel who interact with MORES such as ambulance drivers, administrative staff, and patients being referred within the system could capture more in-depth data beyond what was offered by frontline health workers. Finally, we elected not to utilize a preexisting framework for the conceptualization of our results, given that commonly referenced usability, acceptability, and feasibility metrics for mHealth did not align with the evaluative approach and subsequent immersion in the data. This was apparent, especially for the impact of MORES on health system strengthening. To increase study rigor, we used a validated codebook and 4 separate metrics to ensure accuracy including member checking. However, developing flexible frameworks to better conceptualize usability, effectiveness, and broader benefits of mHealth technologies in LMICs could be highly valuable for future studies.

### Conclusions

The MORES intervention was perceived to have high usability, fidelity, and effectiveness for reducing delays and optimizing referrals in rural Liberia. MORES also demonstrated 6 components of health systems strengthening which positively impacted accountability among health workers, feedback mechanisms, interprofessional dynamics, longitudinal follow-up care, and system-level efficiencies through recording delay factors and electronic health record infrastructure. Findings from this study suggest that MORES demonstrates high feasibility for scaling to other facilities and counties in Liberia to improve maternal and neonatal referral pathways. These conclusions can have direct policy and program implications for supporting MORES funding, implementation, and scaling at a national level. More widely, these findings can be highly useful to policymakers, clinicians, and researchers intending to implement and scale technology-based obstetric referral systems in low-resource contexts to accomplish intended aims, anticipate challenges prior to implementation, and enhance impacts on health system strengthening.

## Appendix

Multimedia Appendix 1 Structured interview script for the usability study among front-line obstetric health workers using Mobile Obstetric Referral Emergency System in rural Liberia.

## Abbreviations

COREQ: consolidated criteria for reporting qualitative research
EHR: electronic health record
LMIC: low- and middle-income country
mHealth: mobile health
MORES: Mobile Obstetric Referral Emergency System
RA: research assistant
RHF: rural health facility
