# Supplementary material for: Implementation of a Technology-Based Mobile Obstetric Referral Emergency System (MORES): Qualitative Assessment of Health Workers in Rural Liberia
Source: JMIR Mhealth Uhealth. 2024 Nov 13;12:e58624. doi: 10.2196/58624 (PMC11602760; doi:10.2196/58624)
Supplement: Multimedia Appendix 1 [file mhealth_v12i1e58624_app1.docx]

Multimedia Appendix 1: Health worker interview script

**Satisfaction Survey**

All end users including midwives/nurses who have used the WAT-RT System were invited to complete the following survey to examine the acceptability, feasibility, usability, and satisfaction with the WAT-RT System. The survey was conducted at the end of the study.

**Facility Name**:  **Participant ID Number**:

**Date**: **Role**:

**Age**: **Professional Service**:

Now I would like to ask you a few questions about the WhatsApp program you have been using.

1. In your own words, tell me the purpose of this WhatsApp program.
2. How well do you think you understand the WhatsApp program?
3. How helpful/useful do you think the application is for you?
4. How helpful/useful do you think the application is for caring for pregnant women?
5. It has been helping us to fight against maternal deaths and neonatal deaths.
6. How often do you think you would use the WhatsApp platform?
7. What is good about the program?
8. What is difficult about using the program?
9. What could make the program better?
10. How much support do you need to use the program?
11. How was WhatsApp helpful to prepare for a referral?
12. How does the program support communication between your clinic and the Referral Hospital?
13. Was feedback from the referral hospital helpful?
14. What was the average time it took you to get feedback from the referral facility?
15. Other comments?
